# Climate Change and Its Impact on Ocular Health: A Systematic Review

**DOI:** 10.7759/cureus.91614

**Published:** 2025-09-04

**Authors:** Nneoma S Onyeze, Joy Jacob

**Affiliations:** 1 Surgery, Ninewells Hospital, Dundee, GBR; 2 Virology, Imperial College London, London, GBR

**Keywords:** air pollution, and eye disease, cataracts, climate change, dry eye syndrome, environmental exposure, ocular health, uv radiation

## Abstract

Climate change has emerged as a pressing public health concern, with growing evidence that its environmental impacts extend to ocular health. Rising ultraviolet (UV) radiation, deteriorating air quality, and extreme weather events contribute to both acute and chronic eye diseases. This systematic review examines existing literature linking climate-related environmental exposures to ocular disorders, focusing on cataract formation, dry eye disease (DED), and meteorologically induced ocular surface instability. It also identifies at-risk populations and research gaps to inform clinical and policy responses. A comprehensive search of peer-reviewed articles published between 2000 and 2024 was conducted using PubMed, Scopus, and Web of Science. Inclusion criteria encompassed original research, epidemiological studies, and reviews that examined the effects of climate change, UV radiation, air pollution, or meteorological variation on human ocular health. Exclusion criteria included studies focused exclusively on indoor risk factors or lacking environmental context.

A total of 18 studies met the inclusion criteria and were thematically synthesized across three core domains: (1) UV exposure and cataracts, (2) air pollution and ocular surface disorders, and (3) meteorological variability and ocular physiology. Evidence indicates strong associations between increased UV-B exposure and elevated cataract risk, particularly in equatorial and high-sunlight regions. Air pollutants - especially PM2.5, ozone, and nitrogen dioxide - were linked to higher prevalence and severity of DED due to tear film instability and surface inflammation. Climatic shifts such as heatwaves and humidity drop further exacerbate ocular surface stress. Vulnerable groups include outdoor workers, elderly individuals, and populations in low-resource or high-pollution regions. Environmental changes driven by climate disruption pose an increasingly recognized threat to ocular health. Addressing this challenge requires multidisciplinary research, public health preparedness, and equitable access to eye care and protective resources.

## Introduction and background

In recent decades, the conversation surrounding climate change has shifted from abstract environmental theory to an immediate public health emergency [1,2]. As global temperatures rise, weather patterns shift unpredictably, and air quality deteriorates, the health implications of a warming planet are becoming increasingly evident [3,4]. While respiratory, cardiovascular, and infectious diseases have dominated the discourse, one critical yet often overlooked area is ocular health. The human eye, by virtue of its constant exposure to the external environment, is a vulnerable target for the cascading effects of climate change [3-5].

Ultraviolet (UV) radiation, now more potent due to stratospheric ozone depletion, is a well-documented risk factor for cataract formation [5,6]. Likewise, the surge in air pollutants - particularly fine particulate matter - has been linked to a growing burden of ocular surface diseases such as dry eye syndrome. Heatwaves and sudden shifts in humidity, once rare, are now regular occurrences that exacerbate tear film instability and trigger inflammatory responses on the eye’s surface [7]. In regions increasingly affected by wildfires, floods, and dust storms, the threat extends beyond chronic irritation to include acute ocular injuries and infections.

The intersection of climate change and ophthalmology is not simply a scientific curiosity - it is an urgent global challenge that demands a multidisciplinary response [8-10]. As researchers continue to unravel the biological pathways affected by environmental changes, clinicians must prepare to recognize and manage emerging ocular presentations. This review aims to synthesize current knowledge on the ocular impacts of climate change, identify populations at heightened risk, and outline preventative strategies that can help safeguard vision in an era of environmental uncertainty [11].

Ultraviolet radiation and cataract formation

One of the most well-established links between climate change and ocular health is the association between UV exposure and cataractogenesis. The depletion of the stratospheric ozone layer has led to increased levels of UV-B radiation reaching the Earth’s surface. Prolonged exposure to UV-B has been implicated in cortical and nuclear cataract formation [11-13]. Epidemiological data from equatorial regions, where solar intensity is naturally higher, consistently report elevated cataract incidence. Climate-induced ozone thinning could expand these high-risk zones, making protective measures such as UV-filtering lenses increasingly essential [14,15].

Air pollution and ocular surface disorders

Urbanization and fossil fuel consumption - both contributors to climate change - have intensified air pollution levels worldwide. Airborne pollutants, including particulate matter (PM2.5 and PM10), nitrogen dioxide, sulfur dioxide, and ozone, have been associated with various ocular surface disorders, most notably dry eye disease (DED) [16]. Pollutants can destabilize the tear film, induce oxidative stress, and promote inflammation on the ocular surface. Furthermore, indoor air quality, often overlooked, plays a significant role in symptom exacerbation, particularly in poorly ventilated, urban living environments [16,17].

Dry eye syndrome and changing meteorological patterns

Beyond pollution, climate change alters meteorological variables - humidity, wind patterns, and temperature - all of which influence tear evaporation rates and ocular comfort. Increasingly frequent heatwaves and lower humidity levels can accelerate tear film breakup, aggravating symptoms of dry eye. Seasonal variability in dry eye prevalence is becoming more pronounced, especially in colder regions experiencing drier winters and hotter summers [18,19]. Patients with existing ocular surface disorders, contact lens wearers, and older adults are disproportionately affected [20].

## Review

Methodology

The systematic review adheres to the standards established by the Preferred Reporting Items for Systematic Reviews and Meta-Analysis (PRISMA 2020), guiding literature search, abstract, and full-text screening, as well as selection of inclusion and exclusion criteria.

Search Strategy

To gather relevant literature, a comprehensive search of peer-reviewed articles published between 2000 and 2024 was performed using PubMed, Scopus, Web of Science, and Google Scholar. The initial search identified 312 articles. After removing 27 duplicates, 285 records were screened by reviewing titles and abstracts, resulting in the exclusion of 190 studies that did not meet the inclusion criteria. The remaining 95 full-text articles were assessed for eligibility by independent reviewers. Seventy-seven articles were excluded using the exclusion criteria stated above. Finally, 18 studies met the inclusion criteria and were included in the qualitative synthesis.

Selection Criteria 

We included peer-reviewed original research, epidemiological studies, and reviews published between 2000 and 2024 that examined human eye outcomes such as cataracts, DED, ocular surface disorders, and climate-related acute eye injuries in relation to UV radiation, outdoor air pollution, or meteorological variation. Studies had to be available in English, while those limited to indoor or occupation-specific risks, lacking a clear environmental exposure, or consisting only of animal or laboratory data, case reports, commentaries, conference abstracts, or non-peer-reviewed work were excluded. Priority was given to well-designed studies that directly addressed the ocular effects of environmental and climate change, though animal findings were considered when they helped explain biological mechanisms. Data were synthesized across three themes: UV exposure and lens changes, air pollution and ocular surface disease, and meteorological influences on eye function and disease progression.

Selection of Studies

After an initial screening of titles and abstracts, two reviewers independently evaluated studies for inclusion in this review according to established inclusion and exclusion criteria. We independently assessed the studies based on the same criteria. Disagreements were resolved through consensus or the involvement of another reviewer.

This review synthesizes existing literature on the relationship between climate change and ocular health, with particular emphasis on cataracts, DED, and other environmentally influenced eye disorders. The objective was to consolidate documented associations while also identifying emerging trends and knowledge gaps, thereby guiding future research priorities and informing public health interventions.

Results

**Figure 1:**
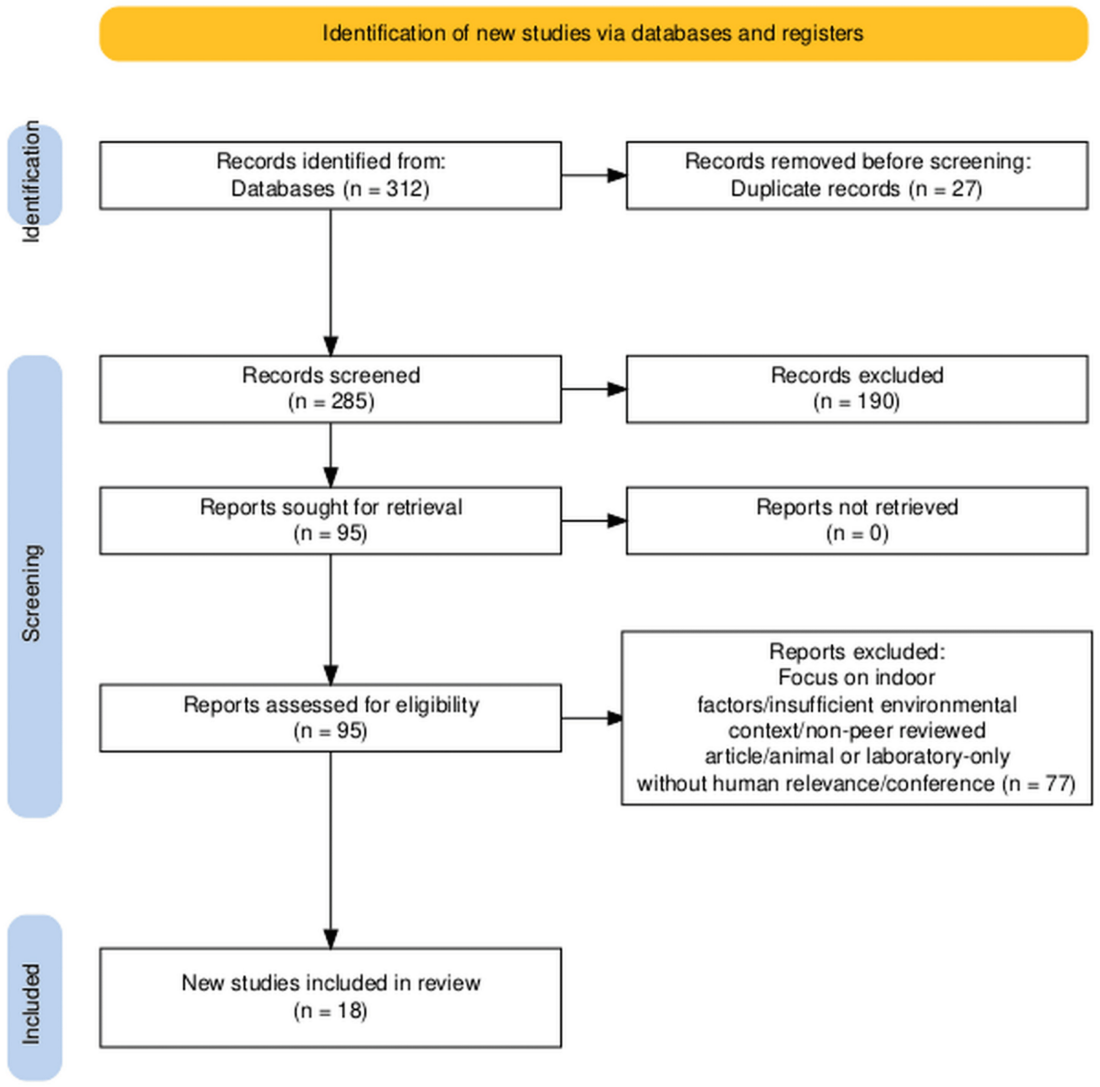
PRISMA flow chart depicting the study selection PRISMA: Preferred Reporting Items for Systematic Reviews and Meta-Analysis [21]

The reviewed literature underscores a clear and growing consensus that climate change exerts a significant influence on ocular health, both directly and indirectly. The two primary ocular conditions consistently linked to climate-related environmental factors are cataracts and DED, each associated with specific environmental exposures such as UV radiation, ambient air pollution, and extreme weather events. Figure 1 presents the PRISMA flowchart of study selection. Table 1 provides a summary of the 18 included studies, and Table 2 details at-risk populations and preventive strategies for climate-related ocular health impacts.

**Table 1:** Summary of 18 studies examining climate-related exposures (UV radiation, air pollution, meteorological variation, environmental stressors) and ocular outcomes Reported outcomes include cataracts, DED, ocular surface instability, and corneal damage, with findings highlighting strong associations and geographic variability AMD: age-related macular degeneration; DED: dry eye disease; UV: ultraviolet

Author	Year	Location	Exposure	Outcome	Key finding										
Echevarría-Lucas et al. [1]	2024	Spain	Air pollutants, occupational factors	Cataract incidence	Analysis showed that long-term exposure to occupational risk factors and ambient air pollution significantly contributes to cataract formation, underscoring the need for protective measures in outdoor workers										
Garg et al. [2]	2024	India	Climate change (UV, air pollution, meteorology)	Cataracts, DED, ocular surface instability	Review highlighted that rising UV radiation, worsening air quality, and meteorological shifts collectively increase risks of cataracts, DED, and ocular surface instability										
Ghosh et al. [3]	2023	India	Climate change, environmental factors	Prevalence of ocular diseases (cataracts, DED, infections)	Narrative review reported that heat, UV, and dust storms in India are directly linked to higher prevalence of dry eye, cataracts, and infectious eye conditions										
Wong et al. [4]	2023	Singapore	Climate change	Range of ocular disorders (cataracts, DED, keratitis)	Scoping review found strong evidence connecting climate change to a spectrum of eye diseases, including cataracts, DED, and infectious keratitis, while highlighting research gaps										
Lucas et al. [5]	2015	Australia/UK	Stratospheric ozone depletion (UV-B)	UV-related cataracts and systemic disorders	The study demonstrated that stratospheric ozone depletion increases UV-B penetration, elevating risk for cataracts and other UV-related disorders, with equatorial populations most affected										
AlRyalat et al. [6]	2022	Jordan	Air pollution, climate change	DED prevalence and ocular surface inflammation	Systematic review concluded that particulate matter, NO₂, and O₃ are consistently associated with increased prevalence and severity of DED and ocular surface inflammation										
Jaggernath et al. [7]	2013	South Africa	UV radiation, water changes	Cataract occurrence, ocular surface irritation	Findings indicated that increased UV radiation and climate-related water scarcity are linked to higher cataract incidence and ocular surface irritation										
Mandell et al. [8]	2020	USA	Air pollution, weather	DED severity	The large cohort study showed that elevated ozone and PM₂.₅ significantly worsen DED symptoms, with older adults most affected during high pollution events										
Kabata et al. [9]	2024	Japan	Seasonal variation, meteorology	Surgical interventions and clinic visits for DED	Data revealed seasonal spikes in DED severity, with winter and early spring showing the highest clinic visits and surgical interventions for dry eye conditions										
Thomas et al. [10]	2012	Australia	Climate change, UV	Cataract risk and other UV-related eye diseases	Review emphasized that interactions between climate change and UV radiation amplify risks for cataracts and other UV-related eye diseases, calling for integrated preventive strategies										
Gorgadze et al. [11]	2023	Georgia	Environmental stressors	Corneal damage and visual system impairment	The study reported that environmental hazards such as dust storms and industrial pollutants disrupt ocular function and increase the risk of corneal damage										
Kansal and Khan [12]	2023	India	Environmental changes	Vision health deterioration (cataracts, DED)	Review underscored that climate-related factors, including UV and particulate pollution, pose growing threats to vision health and require urgent policy intervention										
Millen et al. [13]	2023	USA	Air pollution	Chronic ocular diseases (DED, AMD)	Scoping review linked long-term exposure to PM₂.₅ and NO₂ to chronic ocular diseases such as DED and age-related macular degeneration										
Zhou et al. [14]	2024	China	Air pollution, meteorology	DED symptom severity and prevalence	The epidemiological study showed that exposure to pollutants combined with low humidity and high temperature strongly aggravates DED symptoms and prevalence										
Wong et al. [15]	2021	Singapore	Ophthalmology practices, sustainability	Environmental footprint of ophthalmology	Narrative review stressed the high carbon footprint of ophthalmology practices, advocating for sustainable surgical and clinical approaches to align with climate goals										
Balasubramanian et al. [17]	2020	India	PM2.5	Cataracts, DED, ocular surface inflammation	Review summarized that PM₂.₅ exposure is strongly associated with DED, cataracts, and ocular surface inflammation, particularly in densely polluted urban regions										
McCarty and Taylor [16]	2002	Australia	UV radiation	Cataract development	Epidemiological evidence showed a clear and dose-dependent relationship between UV exposure and cataract development, highlighting the preventive benefits of UV-blocking eyewear										
Yan et al. [18]	2022	China	Meteorological variables	DED prevalence and severity	Meta-analysis concluded that temperature, humidity, and seasonal changes significantly alter DED prevalence, with dry, cold climates increasing symptom burden										

**Table 2:** At-risk populations and preventive strategies for climate-related ocular health impacts LMICs: low- and middle-income countries; UV: ultraviolet

Population	Risk factors	Preventive strategies
Outdoor workers (farmers, construction workers)	High UV exposure, dust, and air pollution	UV-blocking eyewear, hats, hydration, and workplace safety policies
Elderly individuals	Cumulative UV exposure, reduced tear production	Regular eye exams, protective eyewear, and indoor humidification
Urban residents	Air pollution (PM2.5, NO_2_, O_3_)	Air purifiers, reduced outdoor activity on high pollution days
Firefighters/disaster responders	Smoke, dust, debris	Protective goggles, frequent eye irrigation, and occupational safety training
Populations in LMICs	Limited access to eye care/protection	Affordable UV eyewear, improved eye care infrastructure
Contact lens wearers	Dry eye exacerbated by pollution/heat	Lubricating drops, lens hygiene, avoiding lens wear during extreme events

Cataract Development and UV Exposure

**Figure 2:**
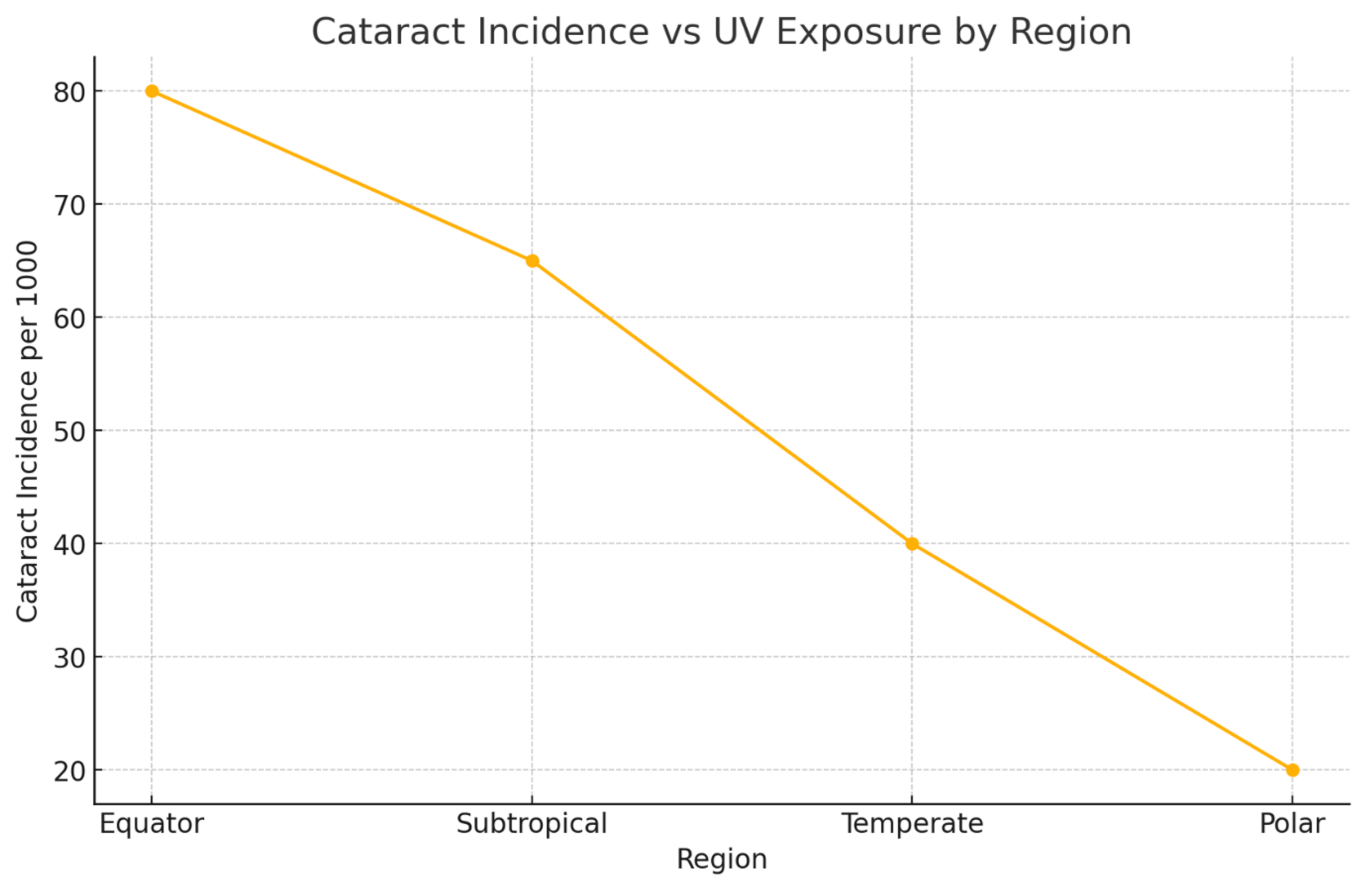
Line graph demonstrating a strong inverse relationship between geographic latitude and cataract incidence, highlighting the role of UV radiation exposure in cataractogenesis Created by authors based on data extracted from epidemiological data reported in studies by Lucas et al. (2015), WHO (2015), and the Blue Mountains Eye Study (Garg et al., 2024). UV: ultraviolet

Numerous studies demonstrate a robust association between increased UV radiation, particularly UV-B, and cataract formation [3-5,17]. The World Health Organization (WHO) estimates that up to 20% of cataract cases globally may be attributable to UV exposure, although the precise proportion varies by geography, study design, and population characteristics [3]. Stratospheric ozone depletion, largely driven by chlorofluorocarbons (CFCs), has resulted in an estimated 1-2% increase in UV-B radiation per decade in some regions, especially those near the poles and at high altitudes [4,5] as illustrated in Figure 2.

Epidemiological studies support a geographic correlation between UV intensity and cataract incidence. For instance, research shows that individuals living closer to the equator have a 60% higher prevalence of cortical cataracts compared to those in higher latitudes [5]. In Australia, which experiences high ambient UV levels, the Blue Mountains Eye Study found that residents exposed to high lifetime sunlight had an odds ratio (OR) of 1.6 for developing nuclear cataracts [4]. Furthermore, UV-blocking interventions have proven effective: Studies indicate that wearing sunglasses that block 99-100% of UVA and UVB rays may reduce the risk of UV-induced cataracts by up to 20%, though this effect is dependent on adherence, duration of use, and other individual factors [12].

In low- and middle-income countries (LMICs), where access to protective eyewear remains limited, the burden is disproportionately higher. As climate change intensifies and stratospheric ozone recovery remains gradual, expanded access to UV protection is a crucial preventive strategy [12].

Dry Eye Disease and Air Pollution

DED has emerged as a major ocular consequence of environmental degradation, particularly in urban centres characterized by persistently poor air quality. Airborne pollutants such as fine particulate matter (PM₂.₅ and PM₁₀), nitrogen dioxide (NO₂), sulphur dioxide (SO₂), and ozone (O₃) are known to destabilize the tear film, induce oxidative stress, and promote inflammation on the ocular surface [6-8,16].

**Figure 3:**
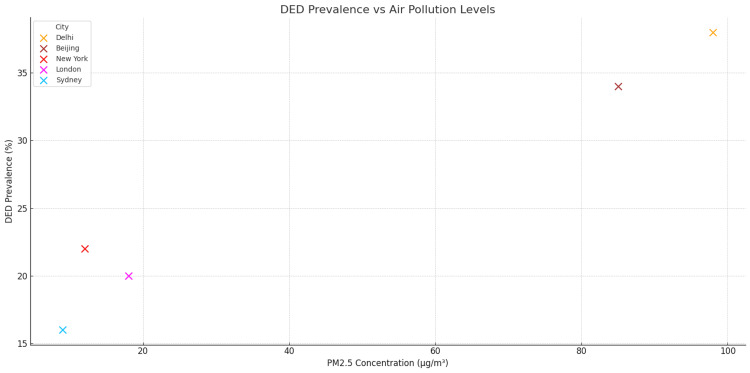
This scatter plot illustrates the relationship between PM2.5 air pollution levels and the prevalence of DED across five major cities Created by authors based on data extracted from [6,7,8] DED: dry eye disease

Quantitative data from multiple studies underscore the strength of this association. A large cross-sectional study in Beijing reported that individuals exposed to PM₂.₅ concentrations exceeding 100 µg/m³ had a 65% increased risk of developing DED symptoms compared to those in lower-exposure areas [7]. In Delhi, one of the world’s most polluted cities, the prevalence of DED was as high as 32.3% among adults, with a strong correlation to ambient PM₁₀ levels [6]. Similarly, in Los Angeles, elevated ozone exposure was associated with a 30% higher incidence of DED in older adults, according to data from Mandell et al. [8]. Further supporting this association, a global review by AlRyalat et al. concluded that for every 10 µg/m³ increase in PM₂.₅, the odds of reporting DED symptoms increased by 13% [6]. The chronicity of exposure is particularly harmful, as prolonged contact with polluted air sustains ocular surface inflammation and disrupts tear film homeostasis, as illustrated in Figure 3.

These findings highlight the urgent need to integrate air quality management into ophthalmologic and public health strategies, particularly in high-density urban areas where populations are chronically exposed to hazardous atmospheric conditions.

Meteorological Extremes and Ocular Surface Stress

Climate change-induced shifts in weather patterns-characterized by extended droughts, sudden humidity changes, and frequent heatwaves-have been shown to exacerbate symptoms in patients with existing ocular surface disease [9,10,18]. High ambient temperatures and low relative humidity increase tear film evaporation, aggravating dry eye symptoms. For example, tear breakup time (TBUT) has been observed to decrease by up to 30% in low-humidity environments (<20%), significantly reducing tear film stability in affected individuals [9].

Seasonal variation in DED has also been well documented. A large-scale Japanese study analysing over 7,000 cases found that DED-related surgeries and clinic visits increased by 15-20% during winter and early spring, with symptoms strongly correlated to cold temperatures, dry air, and indoor heating use [10]. Similarly, an elevated incidence of DED was reported during months with average indoor relative humidity levels as low as 10-15%, particularly in temperate regions [10]. These findings underscore the importance of environmental adaptation in DED management, including maintaining indoor humidity above 30%, wearing protective eyewear outdoors, and avoiding prolonged exposure to dry or extreme weather conditions.

Acute Ocular Injuries From Environmental Disasters

Emerging evidence links climate-related disasters-such as wildfires, floods, and dust storms-to significant spikes in ocular injuries and infections [2,11,13-15,20]. Wildfire smoke, which contains PM₂.₅, carbon monoxide, and volatile organic compounds, has been directly associated with eye irritation and inflammatory ocular conditions. A retrospective study in California reported a 66% increase in emergency eye clinic visits for conjunctivitis and keratitis during the peak of the 2018 Camp Fire, particularly among individuals with pre-existing DED [13]. Similarly, during the 2019-2020 Australian bushfire crisis, a 40% rise in ocular surface disorders was documented among firefighters and outdoor workers exposed to wildfire smoke [11]. Prolonged smoke exposure not only worsens symptoms of dry eye but also increases the incidence of microbial keratitis in high-risk populations.

Floodwaters also pose a serious threat, particularly in disaster-affected or under-resourced regions. A post-disaster surveillance study following the 2018 Kerala floods recorded a threefold increase in conjunctivitis and corneal infections in temporary relief camps, attributed largely to poor hygiene and exposure to contaminated water [14]. Dust storms, especially common in Middle Eastern and North African countries, have been linked to ocular trauma and infection. A study in Riyadh reported a twofold increase in cases of corneal abrasion and allergic conjunctivitis following major dust events [15,20].

At-Risk Populations and Health Disparities

The findings consistently point to increased vulnerability among specific population groups, including outdoor laborers, agricultural workers, the elderly, and residents of equatorial or high-pollution regions [1,2,6]. These groups face higher levels of exposure to UV radiation, airborne pollutants, and meteorological stressors, placing them at disproportionate risk for ocular diseases. For instance, outdoor workers have up to a 2.2-fold increased risk of developing cortical cataracts due to prolonged sun exposure without adequate protective eyewear [1]. In rural India, over 70% of agricultural workers reported not owning UV-blocking sunglasses, and nearly 60% had never undergone an eye examination-despite high rates of ocular complaints such as dryness, irritation, and blurry vision [6].

Older adults also demonstrate heightened vulnerability. Individuals over the age of 60 are 40% more likely to report moderate-to-severe dry eye symptoms when exposed to elevated PM₂.₅ levels, compared to younger adults [2]. These risks are further amplified in LMICs, where healthcare infrastructure is often insufficient. In some LMICs, fewer than 20% of rural clinics are equipped with basic ophthalmic tools such as slit lamps or tonometers [6]. Moreover, the cost and accessibility of protective measures-such as UV-filtering eyewear and indoor air purifiers-remain out of reach for many at-risk populations.

Discussion

The findings of this review reveal a complex and evolving relationship between climate change and ocular health-one that is multifaceted, under-recognized, and increasingly urgent. As environmental stressors intensify, their direct and indirect effects on the eye are becoming more evident, with consequences ranging from discomfort and reduced quality of life to irreversible visual impairment [2,6,12].

Cataracts, already the leading cause of blindness worldwide, are likely to become even more prevalent as UV radiation levels rise due to ongoing stratospheric ozone depletion and persistent greenhouse gas emissions [3-5]. While cataractogenesis is influenced by numerous factors - including genetics, age, and systemic conditions - the contribution of environmental exposure, particularly to UV-B radiation, is substantial and cannot be overlooked. In equatorial and high-sunlight regions, where ambient UV exposure is consistently elevated, cataract incidence is disproportionately high - a trend that may become more widespread as UV levels increase globally [4,5,17].

DED, often dismissed as a minor or cosmetic condition, emerges in this review as a chronic disorder highly sensitive to environmental conditions [6-8,10,16,18]. Long-term exposure to air pollutants and changing meteorological factors-such as low humidity, high wind, and heatwaves-has been shown to destabilize the tear film, induce oxidative stress, and promote persistent ocular surface inflammation [6-10,18,19]. Urban environments characterized by poor air quality-whether due to industrial emissions, vehicular pollution, or wildfire smoke-have reported rising DED prevalence, particularly among individuals with pre-existing ocular surface disorders, advanced age, or high screen exposure [6,8,16]. 

**Figure 4:**
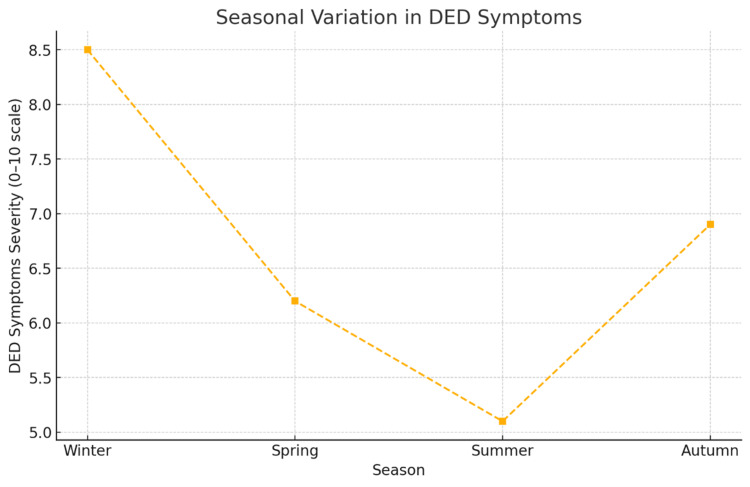
The graph illustrates the seasonal fluctuation in DED symptom severity on a 0-10 scale Data adapted from Kabata et al. (2024) and Mandell et al. (2020) DED: dry eye disease

Furthermore, the rise in extreme weather events-including dust storms, wildfires, and floods-introduces new forms of ocular risk. These events can cause mechanical trauma, exacerbate allergic conjunctivitis, and increase the risk of infectious eye diseases due to exposure to airborne particulates and waterborne microbes [2,11]. Seasonal variation in dry eye symptoms is evident, with peak symptom severity during winter and early spring (Figure 4) (Kabata et al., 2024; Mandell et al., 2020). Despite growing evidence, ophthalmologic preparedness for environmental disasters remains underdeveloped in many regions worldwide [12].

Perhaps most importantly, this review underscores the issue of health equity. The burden of climate-sensitive eye diseases is not evenly distributed. Vulnerable populations-such as individuals in low-income settings, rural communities, and outdoor labour sectors-are often the most exposed yet have the least access to preventive care and treatment [6,12]. Addressing these challenges will require interdisciplinary collaboration. Ophthalmologists, environmental scientists, public health professionals, and policymakers must work together to integrate ocular health into broader environmental health strategies. Preventive measures should include the implementation of community-level educational programs, increased access to UV protection, stricter air quality regulations, and sustainable healthcare delivery-particularly in ophthalmic procedures such as cataract surgery, which themselves contribute to environmental impact [12].

A major limitation of this study is the constraint imposed by the diversity and quality of the available studies, as well as the lack of long-term, population-based research on climate-related eye disease. Evidence was largely drawn from high-income and urban settings, leaving vulnerable low-resource populations underrepresented, which limits the generalizability of the findings.

Study Quality, Relevance, and Gaps 

The body of evidence linking climate change to ocular health outcomes is growing but remains heterogeneous in scope and methodological rigor. The majority of included studies were cross-sectional or retrospective in design, which provides useful associations but limits causal inference. Few longitudinal, population-based studies have been conducted, and this restricts the ability to establish long-term trends or evaluate cumulative exposures such as lifetime UV radiation or chronic air pollution. The reliance on self-reported ocular symptoms, particularly for DED, may also introduce reporting bias, as standardized diagnostic criteria were not consistently applied across studies.

Relevance to global health is clear: cataract and DED are among the most common ocular conditions worldwide, and their exacerbation by environmental stressors underscores a major emerging public health challenge. However, much of the available evidence derives from high-income, urban settings such as Australia, Japan, the United States, and parts of Europe. Studies from LMICs - where both climate change vulnerability and access to ophthalmic care are most pressing - are underrepresented. This geographic imbalance limits generalizability and risks obscuring the disproportionate burden faced by rural and resource-limited populations.

Several important knowledge gaps were identified. First, there is a limited understanding of the biological mechanisms through which pollutants and meteorological extremes interact to accelerate ocular surface disease and lens opacification. Experimental and translational research could clarify these pathways. Second, evidence on acute ocular injuries from climate-related disasters, such as floods and wildfires, is largely anecdotal or confined to small case series; systematic surveillance and large-scale epidemiological data are lacking. Third, protective interventions-such as the effectiveness of UV-blocking eyewear, indoor air filtration, or public health advisories-have not been rigorously evaluated in climate-vulnerable populations.

Finally, health equity remains an underexplored dimension. Populations at highest risk - including outdoor laborers, elderly individuals, and residents of LMICs - are seldom the focus of targeted research, and data on disparities in exposure, protection, and treatment are scarce. Addressing these gaps will require well-designed, multicentre studies with standardized outcome measures, inclusion of vulnerable regions, and interdisciplinary approaches that integrate ophthalmology with environmental and climate sciences.

## Conclusions

Climate change poses a mounting threat to ocular health, with strong evidence linking increased UV radiation, air pollution, and extreme weather to conditions like cataracts and DED. These environmental stressors are not only contributing to rising disease prevalence but are also disproportionately affecting vulnerable populations - such as outdoor laborers, the elderly, and communities in low-resource settings. Despite the growing body of evidence, key knowledge gaps persist, including a lack of long-term, population-level studies and an insufficient understanding of the biological mechanisms by which environmental exposures affect the eye. This review highlights the variability in exposure assessment, outcome measures, and population characteristics, which limits comparability. Climate-related ocular impacts remain under-recognized in public health planning and underrepresented in research funding priorities. Addressing these challenges requires a coordinated, interdisciplinary approach. Practical recommendations include improving access to UV-protective eyewear, integrating eye health into occupational safety standards, enhancing indoor air quality, and strengthening eye care infrastructure in climate-vulnerable regions. Public health messaging should incorporate guidance on protecting eye health during periods of poor air quality or extreme weather. Most critically, more research is needed to inform evidence-based interventions, especially in underserved areas. By positioning vision care as a core component of climate resilience, policymakers and clinicians can help mitigate future burdens and promote equity in the face of environmental change.

## References

[1] Echevarría-Lucas L, Senciales-González JM, Rodrigo-Comino J (2024). Analysing the evidence of the effects of climate change, air pollutants, and occupational factors in the appearance of cataracts. Environments.

[2] Garg P, Shukla R, Shrinkhal S, Singh SP, Banerjee N (2024). Climatic shifts and vision: understanding the impact of climate change on ocular health. Int J Health Sci (Qassim).

[3] Ghosh D, Ghosh S, Mukherjee G (2023). Impact of climate change and related environmental factors on eye health in India - a narrative review. Ecol Environ Conserv.

[4] Wong YL, Wong SW, Ting DSJ (2023). Impacts of climate change on ocular health: a scoping review. J Clim Change Health.

[5] Lucas RM, Norval M, Neale RE, Young AR, de Gruijl FR, Takizawa Y, van der Leun JC (2015). The consequences for human health of stratospheric ozone depletion in association with other environmental factors. Photochem Photobiol Sci.

[6] Alryalat SA, Toubasi AA, Patnaik JL, Kahook MY (2024). The impact of air pollution and climate change on eye health: a global review. Rev Environ Health.

[7] Jaggernath J, Haslam D, Naidoo K (2013). Climate change: impact of increased ultraviolet radiation and water changes on eye health. Health.

[8] Mandell JT, Idarraga M, Kumar N, Galor A (2020). Impact of air pollution and weather on dry eye. J Clin Med.

[9] Kabata Y, Terauchi R, Nakano T (2024). Seasonal variations and environmental influences on dry eye operations in Japan. Sci Rep.

[10] Thomas PO, Swaminathan A, Lucas RM (2012). Climate change and health with an emphasis on interactions with ultraviolet radiation: a review. Glob Change Biol.

[11] Gorgadze G, Giorgobiani M, Jikurashvili T (2023). Impact of harmful environmental factors on the visual system. Georgian Sci.

[12] Kansal K, Khan H (2023). Environmental factors and eye health: protecting your vision in a changing world. Int J Ophthalmol Optom.

[13] Millen AE, Dighe S, Kordas K, Aminigo BZ, Zafron ML, Mu L (2024). Air pollution and chronic eye disease in adults: a scoping review. Ophthalmic Epidemiol.

[14] Zhou HZ, Liu X, Zhou D (2024). Effects of air pollution and meteorological conditions on DED: associated manifestations and underlying mechanisms. Klin Monbl Augenheilkd.

[15] Wong YL, Noor M, James KL, Aslam TM (2021). Ophthalmology going greener: a narrative review. Ophthalmol Ther.

[16] McCarty CA, Taylor HR (2002). A review of the epidemiologic evidence linking ultraviolet radiation and cataracts. Dev Ophthalmol.

[17] Balasubramanian SA, Sharma T, Raman R (2020). Exposure to PM2.5 and its association with ocular diseases: a review. Int J Environ Res Public Health.

[18] Yan Z, Chen Q, Xu J (2022). Meteorological variables and dry eye disease: a systematic review and meta-analysis. Ophthalmic Epidemiol.

[19] Davis HE, Wilson J, Patel R (2020). Heatwaves, hydration, and eye health: emerging trends. Environ Health Insights.

[20] Mehta JS, Gupta N, Banerjee R (2008). Corneal effects of ambient pollution and environmental stress. Cornea.

[21] Haddaway NR, Page MJ, Pritchard CC, McGuinness LA (2022). PRISMA2020: An R package and Shiny app for producing PRISMA 2020-compliant flow diagrams, with interactivity for optimised digital transparency and Open Synthesis. Campbell Syst Rev.

